# Light-responsive transcription factor ApTrihelix1 modulates andrographolide biosynthesis via targeting *ApCPS2* in *Andrographis paniculata*

**DOI:** 10.1093/hr/uhag118

**Published:** 2026-04-07

**Authors:** Ling Zhang, Hua Yang, Tingting Jing, Yufang Hu, Siqing Fan, Shuyun Tian, Xuesong Wang, Honglei Jin, Wei Sun, Mingkun Huang

**Affiliations:** Jiangxi Provincial Key Laboratory of Ex Situ Plant Conservation and Utilization, Lushan Botanical Garden, Chinese Academy of Sciences, 9 Zhiqing Road, Jiujiang, Jiangxi 332900, China; Jiangxi Provincial Key Laboratory of Ex Situ Plant Conservation and Utilization, Lushan Botanical Garden, Chinese Academy of Sciences, 9 Zhiqing Road, Jiujiang, Jiangxi 332900, China; Jiangxi Provincial Key Laboratory of Ex Situ Plant Conservation and Utilization, Lushan Botanical Garden, Chinese Academy of Sciences, 9 Zhiqing Road, Jiujiang, Jiangxi 332900, China; Jiangxi Provincial Key Laboratory of Ex Situ Plant Conservation and Utilization, Lushan Botanical Garden, Chinese Academy of Sciences, 9 Zhiqing Road, Jiujiang, Jiangxi 332900, China; Jiangxi Provincial Key Laboratory of Ex Situ Plant Conservation and Utilization, Lushan Botanical Garden, Chinese Academy of Sciences, 9 Zhiqing Road, Jiujiang, Jiangxi 332900, China; Jiangxi Provincial Key Laboratory of Ex Situ Plant Conservation and Utilization, Lushan Botanical Garden, Chinese Academy of Sciences, 9 Zhiqing Road, Jiujiang, Jiangxi 332900, China; Key Laboratory of Chinese Medicinal Resource from Lingnan, Ministry of Education, School of Pharmaceutical Sciences, Guangzhou University of Chinese Medicine, Guangzhou 510006, China; Key Laboratory of Chinese Medicinal Resource from Lingnan, Ministry of Education, School of Pharmaceutical Sciences, Guangzhou University of Chinese Medicine, Guangzhou 510006, China; Key Laboratory of Chinese Medicinal Resource from Lingnan, Ministry of Education, School of Pharmaceutical Sciences, Guangzhou University of Chinese Medicine, Guangzhou 510006, China; State Key Laboratory for Quality Ensurance and Sustainable Use of Dao-di Herbs Institute of Chinese Materia Medica, China Academy of Chinese Medical Sciences, Beijing 100070, China; Key Laboratory of National Forestry and Grassland Administration for Chinese Herbal Medicine, Institute of Chinese Materia Medica, China Academy of Chinese Medical Sciences, Beijing 100070, China; Jiangxi Provincial Key Laboratory of Ex Situ Plant Conservation and Utilization, Lushan Botanical Garden, Chinese Academy of Sciences, 9 Zhiqing Road, Jiujiang, Jiangxi 332900, China

## Abstract

Andrographolide (AD) is the major bioactive component in the Chinese medicinal plant *Andrographis paniculata* (*A. paniculata*), which is widely used for its anti-inflammatory and antiviral properties. In this study, we observed that light is a critical environmental factor for AD biosynthesis and identified a potential transcription factor, *ApTrihelix1,* as a regulator of AD biosynthesis via RNA-seq combined with co-expression analysis. *ApTrihelix1* is light-induced, and knocking down its expression downregulates AD content as well as the expression of several AD biosynthesis-related genes (ADRGs). Furthermore, through high-throughput sequencing methods (e.g. ChIP-seq) and molecular experiments (e.g. Yeast One Hybrid assay), ApTrihelix1 was found to bind to the promoter of *ApCPS2* (a key ADRG) to activate its expression. Taken together, these data strongly support that light upregulates the expression of certain transcription factors (e.g. *ApTrihelix1*), thereby activating key ADRGs (e.g. *ApCPS2*) and promoting AD accumulation in *A. paniculata*.

## Introduction

*Andrographis paniculata* (*A. paniculata*), belonging to the Acanthaceae family, is an important herbaceous medicinal plant widely used for its anti-inflammatory and antiviral properties in China, India, and other Southeast Asian countries [1–4]. *A. paniculata* has been included in the selected medicinal plants of WHO monographs due to its specific accumulation of biologically active ent-labdane-type diterpenoids [5]. Andrographolide (AD) is one of the valuable diterpenoid compounds with pharmaceutical bioactivity in *A. paniculata*, which has only been found to accumulate specifically in this medicinal plant to date [1, 2].

Similar to other secondary metabolites such as artemisinin [6, 7], AD is easily influenced by environmental factors, including both abiotic and biotic ones. Among these, light is an essential environmental factor throughout the entire plant lifecycle [8]. As reported previously, AD accumulation is affected by different light qualities, and recently, a key DOF transcription factor (TF) has been cloned that is able to regulate AD biosynthesis in response to light [9, 10]. Additionally, our preliminary data showed that AD accumulation differed between light-grown and dark-grown *A. paniculata* seedlings. These observations suggest that light is one of the key factors capable of regulating AD biosynthesis.

However, how light regulates AD biosynthesis has remained elusive. Thus, in this study, we identified a key TF, named *ApTrihelix1*, which is co-expressed with *ApCPS2*, a key gene involved in AD biosynthesis. The expression of both *ApTrihelix1* and *ApCPS2* is light-induced. Molecular evidence, such as Yeast One Hybrid and chromatin immunoprecipitation sequencing (ChIP-seq) assays, showed that ApTrihelix1 can bind to the promoter of *ApCPS2* and affect its expression, as well as AD content. Taken together, we provide a molecular framework elucidating how light activates AD accumulation via TFs to promote the expression of ADRGs in *A. paniculata*.

## Results

### Light affects AD accumulation and gene expression in *A. paniculata* seedlings

**Figure 1:**
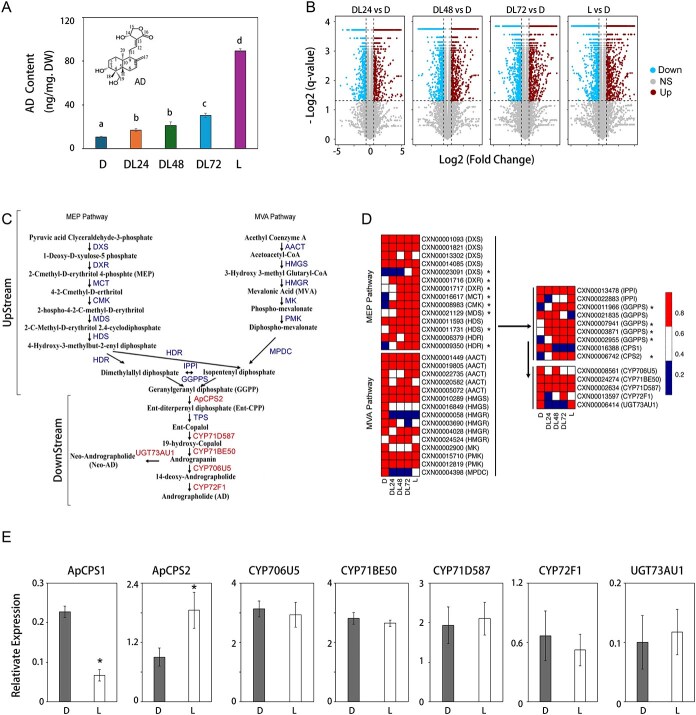
Light affects AD accumulation and the expression of ADRGs in *A. paniculata* seedlings. (A) AD content in *A. paniculata* seedlings grown under different light conditions. D: dark-grown seedlings; L: light-grown seedlings; DL (dark transferred to light): dark-grown seedlings transferred to light conditions for 24 h (DL24), 48 h (DL48), and 72 h (DL72). DW: dry weight. The chemical structure of AD is shown in the top-left corner. Significant differences are marked with a, b, c, and d (ANOVA with Tukey’s test, *P* < 0.05). (B) RNA-seq analysis showing DEGs in DL24, DL48, DL72, and L seedlings compared to D seedlings. Down: downregulated DEGs; NS: genes with no significant change; Up: upregulated DEGs. DEGs were defined as genes with a 1.5-fold change and *q* < 0.05. (C) Overview of the AD biosynthesis pathway. Key enzymes marked in red text have been experimentally validated in *A. paniculata*, while enzymes marked in blue have been functionally characterized in other species. The downstream enzymes are sufficient to complete the entire catalytic process of AD *in vivo*. (D) Heatmap of relative expression levels of genes involved in the AD biosynthesis pathway. The mean FPKM value of three biological replicates was normalized to the maximum FPKM value. Asterisks (^*^) mark genes significantly upregulated in at least one light exposure treatment compared to D. (E) qPCR analysis of several genes in the downstream of the AD biosynthesis pathway, including *ApCPS1*, *ApCPS2, CYP706U5, CYP71BE50*, *CYP71D587*, *CYP72F1*, and *UGT73AU1* under different light conditions. D: dark-grown seedlings. L: light-grown seedlings. Asterisks (*) indicate significant differences between dark (D)- and light (L)-grown *A. paniculata s*eedlings (Student’s *t-*test, *P* < 0.05).

Consistent with our preliminary observations that light affects AD accumulation, to further confirm this, we measured AD content in dark-grown *A. paniculata* seedlings and those subjected to a series of light treatments (Fig. 1A). The results showed that AD content in dark-grown seedlings (D) was ~10.6 ng/mg DW. When transferred to light for 24 h (DL24), 48 h (DL48), and 72 h (DL72), AD content in dark-grown seedlings increased with increasing light exposure time, reaching 16.7, 21.2, and 30.2 ng/mg DW in DL24-, DL48-, and DL72-treated seedlings, respectively. Additionally, fully light-grown (L) seedlings exhibited the highest AD content (~89.4 ng/mg DW) (Fig. 1A), indicating that light is essential for AD biosynthesis in *A. paniculata*.

To further investigate how gene expression changes in response to light exposure, we performed RNA-seq (Fig. S1; Table S1) on seedlings subjected to the corresponding light treatments. Approximately 14.7 to 20.6 M raw reads were obtained and mapped to the *A. paniculata* reference genome (Table S1) [11]. Compared to D seedlings, 3708 (1616 downregulated/2092 upregulated), 4830 (2157/2673), 5427 (2361/3066), and 5523 (2657/2866) differentially expressed genes (DEGs) were identified in DL24, DL48, DL72, and L seedlings (Fig. 1B; Table S2), respectively. Furthermore, GO enrichment analysis (Fig. S2) of these DEGs revealed significant enrichment of several light-related terms, such as ‘Chloroplast organization’ (GO:0009658), ‘Photosynthesis’ (GO:0015979), and ‘Response to abiotic stimulus’ (GO:0009628), indicating that genes responding to light undergo substantial changes in *A. paniculata* seedlings under different light exposure treatments.

AD belongs to the diterpene family, whose metabolism and biosynthesis largely involve two major pathways: the MEP and MVA pathways (Fig. 1C) [1, 11–15]. We examined the expression patterns of AD biosynthesis-related genes (ADRGs) using these RNA-seq data. As shown in Fig. 1D, several genes in the MEP pathway exhibited significant changes during light treatments, whereas genes in the MVA pathway showed no obvious changes (Fig. 1D; Table S2). This indicates that light affects AD biosynthesis primarily by regulating the genes in the MEP pathway rather than the MVA pathway.

Additionally, recent studies have reported that the downstream AD biosynthesis pathway includes *CPS* (*ApCPS1* and *ApCPS2*), *TPS*, and four *CYPs* (Fig. 1C) [11, 14, 15]. Both RNA-seq data and subsequent real-time PCR results showed that only *ApCPS2* exhibited a significantly upregulated expression pattern during light treatments (Fig. 1E), indicating that *ApCPS2* upregulation is a key step in light-induced AD accumulation.

**Figure 2:**
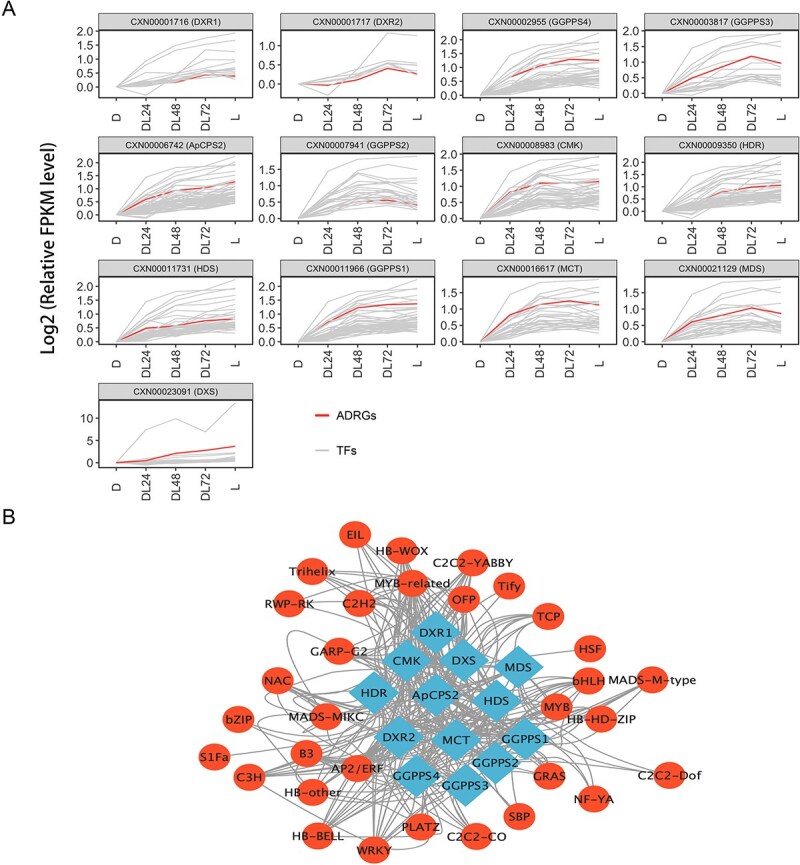
Co-expression analysis of differentially expressed ADRGs and transcription factors (TFs). (A) Relative expression patterns of differentially expressed ADRGs and TFs. L: light-grown seedlings; DL (dark transferred to light): dark-grown seedlings transferred to light conditions for 24 h (DL24), 48 h (DL48), and 72 h (DL72). Pairs of ADRGs and TFs with PCC > 0.9 and *P* < 0.05 were considered co-expressed. (B) Putative regulatory network constructed based on the co-expression analysis described in (A).

To identify TFs that regulate ADRGs in response to light, we performed co-expression analysis by calculating Pearson correlation coefficient (PCC) values between 13 differentially expressed ADRGs (Fig. 1D) and differentially expressed TFs. In total, 406 TF-ADRG pairs with a PCC > 0.90 and *P*-values < 0.05 were identified (Fig. 2A; Table S3), forming a potential regulatory network with these differentially expressed ADRGs (Fig. 2B). These TFs included AP2/ERF, bHLH, Trihelix, and WRKY family members (Fig. S3), which are thought to play important roles in regulating AD biosynthesis. Among them, bHLH, Trihelix, NF-Y, and EIL TFs have been reported to exert light-related regulatory functions in other plants, making them candidates for subsequent experimental investigation.

### ApTrihelix1 regulates AD biosynthesis by modulating ADRG expression

**Figure 3:**
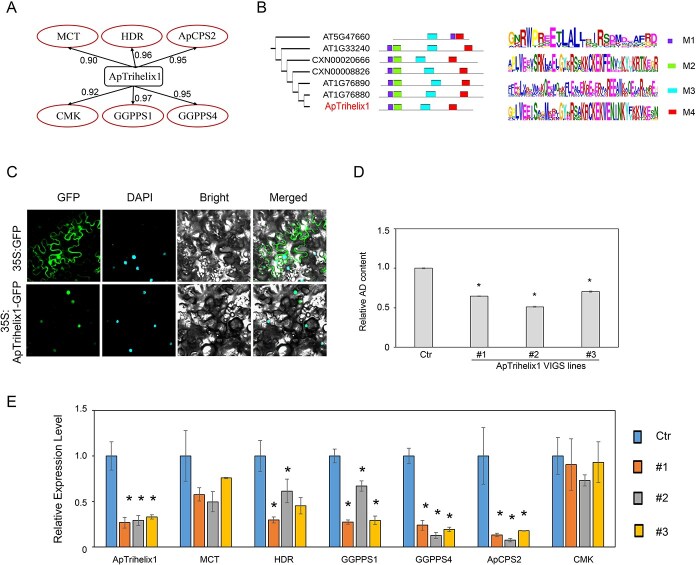
ApTrihelix1 is a key regulator of AD biosynthesis. (A) The co-expressed ADRG partners of ApTrihelix1. Numbers indicate the PCC values. (B) Conserved protein motifs (M1–M4) of ApTrihelix1 and other Trihelix TFs from *Arabidopsis thaliana* and *A. paniculata*. (C) Subcellular localization of ApTrihelix1. Confocal microscopy images of tobacco epidermal cells expressing 35S: GFP (empty vector control) and ApTrihelix1-GFP fusion protein. DAPI: 4′,6-diamidino-2-phenylindole, a nuclear dye, is used as a positive control. Merged: GFP fluorescence was overlaid on bright-field images. Scale bars = 50 μm. (D) Relative AD content in control plants (Ctr) and three *ApTrihelix1 VIGS lines* (#1, #2, and #3). Asterisks (^*^) indicate significant differences between VIGS lines and the control group (*P* < 0.05, Student’s *t*-test). (E) qPCR analysis of relative expression levels of *ApTrihelix1* and six co-expressed ADRGs (*MCT*, *HDR*, *GGPPS1*, *GGPPS4*, *ApCPS2*, and *CMK*) in control plants and three ApTrihelix1 VIGS lines (#1, #2, and #3). Asterisks (^*^) indicate significant differences between VIGS lines and the control group (*P* < 0.05, Student’s *t*-test).

Among these co-expressed TFs, we identified a Trihelix TF, designated *ApTrihelix1* (CXN00007305), which showed co-expression with several differentially expressed ADRGs (Fig. 3A; Figs S4, S5), especially genes involved in downstream steps, such as *ApCPS2* and *GGPPS*. ApTrihelix1 belongs to the GT-2 subfamily (Fig. 3B) and localizes to the nucleus, similar to its homologs (Fig. 3C), as reported previously. As *ApCPS2*, expression of *ApTrihelix1* is light induced (Fig. S6), and these two genes also display a similar expression pattern in different tissues (Fig. S7).

To confirm its role in regulating the AD biosynthesis, we performed the virus-induced gene silencing (VIGS) experiment. We cloned a 300-bp fragment of ApTrihelix1 into the pTRV_156 vector and performed *Agrobacterium*-mediated vacuum infiltration to transform into the 10-day-old seedlings of *A. paniculata* and grown for another 10 days in plant growth chamber. As expected, the AD content in the ApTrihelix1 VIGS lines (#1, #2, and #3) was significantly decreased compared to the control plants (Fig. 3D). Consistent with the decrease of AD content, several *ApTrihelix1* co-expressed ADRGs, such as *ApCPS2* and *GGPPSs*, significantly downregulated in the VIGS lines (Fig. 3E). These data supported that ApTrihelix1 is able to regulate the AD biosynthesis by regulating the expression of ADRGs (e.g. *ApCPS2*).

### Identifying ApTrihelix1 downstream target genes via ChIP-seq method

As TFs can regulate thousands of downstream genes [16], we performed ChIP-seq to genome-wide identification of potential ApTrihelix1 target genes, especially ADRGs that exhibited downregulation in *ApTrihelix1* VIGS lines. We transiently expressed ApTrihelix1 fused with GFP at the C-terminus in *A. paniculata* seedlings via vacuum infiltration, followed by ChIP-seq [17] using the anti-GFP antibody.

**Figure 4:**
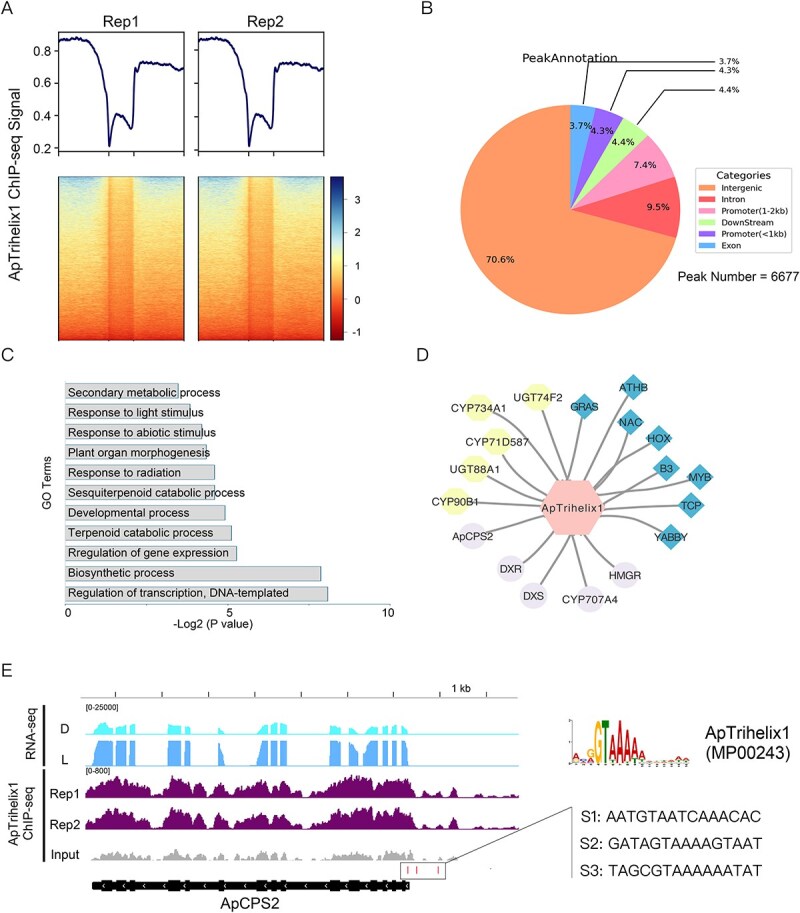
ApTrihelix1 ChIP-seq data summary. (A) ApTrihelix1 ChIP-seq signal profile of two replicates. Rep1 and Rep2 indicate the two ChIP-seq replicates. (B) Genomic annotation of 6677 ApTrihelix1-enriched ChIP-seq peaks. (C) GO enrichment analysis of genes associated with ApTrihelix1 ChIP-seq enriched peaks. The bars on the *x*-axis represent the *P* values of GO enrichment, expressed as −Log₂(*P* value). The label text above each bar indicates the name of the corresponding GO term. (D) A network displaying representative examples of ApTrihelix1-targeted genes. Squares represent transcription factors; Circles represent important secondary metabolic genes or ADRGs. (E) IGV visualization of ApTrihelix1-enriched signals at *ApCPS2* locus. The location of the ApTrihelix1 motif (MP00243) and its corresponding sequences in the *ApCPS2* are shown in the right panel.

We obtained 75–100 M raw reads from two ChIP-seq libraries and one input library (Table S1), with an average overall mapping rate of over 95% to the reference genome. The two ChIP-seq replicates showed a correlation coefficient of 0.64 (Fig. S8) and similar enrichment patterns flanking gene bodies (Fig. 4A). After calling peaks using MACS2 [18], we identified 13 521 and 16 018 enriched peaks in the two ChIP-seq experiments (Table S1), respectively. Among these, a total of 6677 overlapping peaks between the two replicates with a peak width range from 100–1000 bp (Fig. S9) were further considered as potential ApTrihelix1 binding sites (Fig. 4B). Genomic annotation revealed that ~11.7% and 3.7% of these peaks are located in promoter and exon regions (Fig. 4B), respectively.

Gene association analysis identified 3360 genes linked to ApTrihelix1-enriched peaks (Table S4). GO enrichment analysis (Fig. 4C) of these associated genes revealed several relevant terms, such as ‘secondary metabolic process’, ‘response to light stimulus’, and ‘regulation of transcription’, suggesting that ApTrihelix1 plays a regulatory role in transcription and secondary metabolism. For example, ChIP-seq results showed that several TFs (e.g. *NAC*, *B3*, *MYB*) and secondary metabolism-related genes (e.g. *CYP*, *UGT*, *DXR*, *DXS*) are targets of ApTrihelix1 (Fig. 4D; Fig. S10; Table S5). Importantly, *ApCPS2*, an ADRG co-expressed with *ApTrihelix1*, was also identified as a potential target. Scanning the *ApCPS2* promoter sequence revealed three conserved sequences matching the ApTrihelix1 motif (Motif ID: MP00243) (Fig. 4E). These data suggest that ApTrihelix1 might bind to the conserved motif in the *ApCPS2* promoter to regulate its expression.

### Validation of ApTrihelix1 binding to the *ApCPS2* promoter

To avoid false positive signals from ChIP-seq indicating ApTrihelix1 binding to the *ApCPS2*, we performed several validation experiments, including ChIP-qPCR, Y1H assay, and transient transcriptional activation assay.

**Figure 5:**
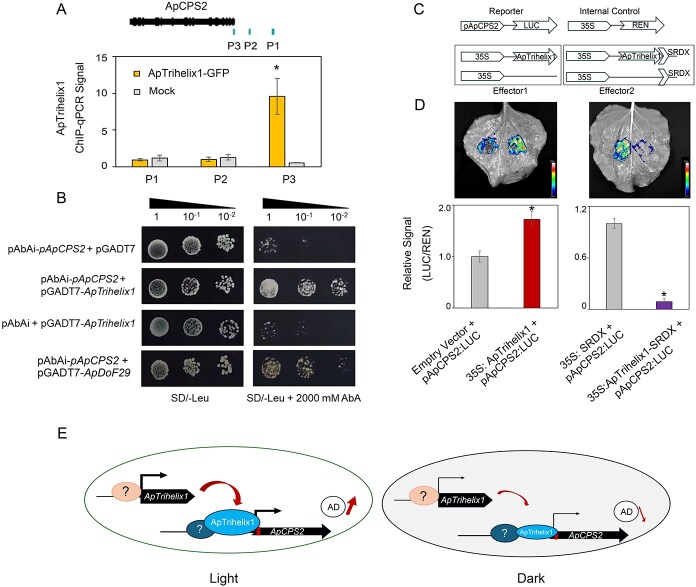
Validation of ApTrihelix1 binding to the *ApCPS2* promoter (*pApCPS2*). (A) ChIP-qPCR confirmed that ApTrihelix1 binds to the P3 region of *pApCPS2*. Enriched ChIP signals represent the qPCR signal of immunoprecipitated (ChIPed) DNA relative to input DNA. *ApACT* was used as the internal control. Asterisks (^*^) indicate significant enrichment of ChIPed signal between the ApTrihelix1-GFP transient transform and the mock plant (Student’s *t*-test, *P* < 0.05). (B) Yeast One-Hybrid (Y1H) assays demonstrated that ApTrihelix1 binds to *pApCPS2*. The interaction between *pApCPS2* and ApDof29 was used as the positive control. (C) Schematic diagram of vector construction for transient expression assays. LUC: firefly luciferase; REN: Renilla luciferase; SRDX: SUPERMAN Repression Domain X (a transcriptional inhibitory domain). (D) Transient expression assays showed that ApTrihelix1 activates *pApCPS2* expression. Upper panel: LUC signal images; lower panel: relative luciferase activity (LUC/REN ratio). Asterisks (^*^) indicate significant differences compared to the empty vector groups (Student’s *t*-test, *P* < 0.05). (E) A brief regulatory model of ApTrihelix1 regulating *ApCPS2* expression. Left panel: under light conditions, *ApTrihelix1* expression is induced by light, producing more protein that binds to the *ApCPS2* promoter, promoting its expression and thereby facilitating AD biosynthesis. Under dark conditions, *ApTrihelix1* expression is low, downregulating *ApCPS2* expression and thus inhibiting AD biosynthesis. Question marks (?) indicate that some unknown transcription factors are involved in this regulation and require further verification.

As shown in Fig. 5A, we designed three primer pairs targeting regions near the conserved ApTrihelix1 motifs in the *ApCPS2* promoter (*pApCPS2*) region, which is defined as the first exon and the 1 kb upstream region of the transcription start site. ChIP-qPCR was conducted using ChIP-enriched DNA from *ApTrihelix1* transient overexpression lines and mock-treated plants. Consistent with ChIP-seq data, ChIP-qPCR signals in the P3 region exhibited significant enrichment compared to the mock group.

For the Y1H assay (Fig. 5B), we cloned the *pApCPS2*, into the *pAbAi* vector and the full-length *ApTrihelix1* coding sequence into the *pGADT7* vector. As expected, ApTrihelix1 bound to *pApCPS2* and activated the reporter’s resistance to Aureobasidin A (AbA), confirming the TF-promoter interaction.

We further constructed a series of reporter and effector vectors for transient transcriptional activation assays (Fig. 5C). When *35S:ApTrihelix1* was co-infiltrated with the reporter (*pApCPS2: LUC*) into tobacco leaves, a significant activation effect was observed compared to the control group (empty vector + reporter) (Fig. 5D). Additionally, we fused the transcriptional repressor domain SRDX to the C-terminus of ApTrihelix1 to generate the *35S:ApTrihelix1-SRDX* effector vector (Fig. 5C). Co-infiltration of this vector with the reporter resulted in a significant inhibitory effect on LUC activity (Fig. 5D). These results demonstrate that *ApTrihelix1* can directly activate the transcriptional activity of the *ApCPS2* promoter. In addition, we observed that the ApTrihelix1 protein level did not gradually decrease with dark treatment, suggesting that light does not affect the accumulation of ApTrihelix1 protein (Fig. S11).

Taken together, these data support a regulatory model (Fig. 5E) wherein *ApTrihelix1* functions as a key regulator governing AD biosynthesis by activating *ApCPS2* and potentially other ADRGs.

## Discussion

Light is a crucial environmental factor for plants’ energy source, growth and development, and stress response [8], and it can also regulate plants’ secondary metabolism. In this study, we previously found that the accumulation of AD in *A. paniculata* is dependent on light. Through different light treatment experiments combined with transcriptome data, we discovered that light could affect gene expression in *A. paniculata*, especially the ADRGs, suggesting that light regulates AD production through the plant’s transcriptional regulatory network. Furthermore, via co-expression analysis, ChIP-seq, Y1H, and other experiments, we identified that ApTrihelix1 can bind to the promoter region of *ApCPS2* to regulate its expression and thus AD synthesis. These data indicate that ApTrihelix1 is one of the key TFs in the light-regulated AD production process.

AD is one of the most valuable bioactive compounds in *A. paniculata* [1] and an important indicator for evaluating *A. paniculata* varieties. However, the content of AD in plants is relatively low, so breeding lines with high AD content is a key breeding goal. Currently, however, molecular breeding research on *A. paniculata* is relatively scarce, especially regarding the transcriptional regulatory molecular mechanisms of AD in plants. Recently, some important TFs such as Dof have been reported to bind to the *CPS2* promoter of *A. paniculata* and regulate AD synthesis in response to light [9, 10]. The MYC-HSFB2b transcription module regulates AD biosynthesis by binding to the *ApCPS1* promoter in response to temperature [19]. ApPIF1 (light signaling) and ApWRKY40 (SA signaling) directly activate key biosynthetic genes *ApGGPPS2* and *ApCPS2*, respectively, and physically interact to integrate light and SA signals, regulating AD production [20]. Epigenetic factors, such as histone modification and chromatin accessibility, have also been reported to participate in the transcriptional regulation of AD and the ADRGs [9, 10, 21]. High light enhances AD accumulation in *A. paniculata* by elevating salicylic acid (SA) levels, and exogenous SA further promotes this biosynthesis. Single-cell RNA-seq and MSI reveal that *ApCPS2*, a key AD biosynthesis gene, is specifically highly expressed in mesophyll cells, which are the main sites of light-induced AD production in *A. paniculata*. Light-responsive TFs ApHY5 and ApPIF1 are co-expressed with *ApCPS2* in mesophyll cell clusters and directly activate *ApCPS2* transcription, forming a light-dependent regulatory module that promotes AD accumulation [22]. These studies suggest that the transcriptional regulation of AD is relatively complex, especially the relationships between these factors. For example, whether there is protein interaction between ApTrihelix1 and other TFs or epigenetic regulators to regulate AD expression still requires further investigation.

In this study, we found that ApTrihelix1 can positively regulate AD biosynthesis, indicating that this gene is an important target site for subsequent molecular breeding. For example, combined with DNA resequencing of population resources, we can screen lines with upregulated genes due to promoter variations of ApTrihelix1. Alternatively, if transgenic technology in *A. paniculata* is permitted for commercial use like some crops, overexpressing *ApTrihelix1* through transgenic technology is another approach for increasing the AD content in plants. Additionally, apart from the ApTrihelix1 TF identified in this study, co-expression data and motif scanning results suggest there are many potential TFs acting as the molecular targets, such as EIL and bHLH (Table S5), but this requires further research.

Additionally, it has been confirmed that AD can be induced by light in this study. Therefore, it may be feasible to further improve AD yield by equipping greenhouses with artificial light sources such as LEDs to optimize light conditions during *A. paniculata* cultivation [23, 24]. Of course, these artificial light sources need to be further optimized for light intensity, photoperiod, and light quality before practical application. Thus, these light quality parameters are also important directions for future research in the culture of *A. paniculata* under greenhouse conditions.

## Conclusion

Overall, our study reveals the regulatory role of ApTrihelix1 TFs regulating the AD biosynthesis via binding to the *ApCPS2* in a light-induced manner.

## Materials and methods

### Plant growth and light treatment

After surface sterilization with 75% ethanol, *A. paniculata* seeds were sown in peat soil (10–15 seeds per pot) and maintained in a greenhouse at 25°C–28°C. Pots were covered with aluminum foil to exclude light for 7 days to facilitate the growth of etiolated seedlings, which were then transferred to normal growth conditions for dark-to-light (D-to-L) treatment for 24, 48, and 72 h. Etiolated seedlings or seedlings grown under normal light conditions for 7 days were used as the dark-grown and light-grown samples, respectively. Samples were then used for AD measurement or RNA extraction for RNA-seq.

### Andrographolide measurement

AD [9, 10] measurement was conducted according to previous reports. Briefly, 0.05 g of fresh seedlings was powdered in liquid nitrogen and AD was extracted with 1.8 ml of methanol. The resulting sample solution was filtered through a 0.45-μm nylon membrane prior to HPLC analysis. Chromatographic separation was performed on a Waters e2695 HPLC system using a Zorbax SB-C18 column (4.6 × 150 mm, 5 μm) at 25°C at the following program: 0–8 min, 21%–25% A; 8–20 min, 25%–28% A; 20–25 min, 28%–85% A; 25–30 min, 85% A. A final concentration of 1 mg/ml AD standard (Shanghai Yuanye Biotechnology Co., Ltd, China) was used to measure the AD content of each sample.

### RNA-seq and data processing

Total RNA of about 0.5 g *A. paniculata* seedlings sample was extracted using the RNeasy Plant Mini RNA Isolation Kit (QIAGEN). A total of 5 μg RNA was used for RNA-seq library preparation. In brief, the mRNA was extracted using the magnetic Dynabeads™ Oligo(dT)_25_ (Invitrogen) following the manufacturer’s instructions. The RNA-seq library was conducted following several steps, including mRNA fragmentation, first-strand cDNA synthesis, second-strand dsDNA synthesis, end repairing, dA tailing, Y-shaped adapter ligation, and final library amplification. The RNA-seq library was sequenced using the Illumina NovaSeq 6000 platform in the paired-end mode. Low-quality adaptors and reads were removed by the TrimGalore software (http://github.com/FelixKrueger/TrimGalore) and then mapped to the *A. paniculata* reference genome [11] using HISAT2 [25], and DEGs were calculated using the Cuffdiff software (http://cole-trapnell-lab.github.io/cufflinks/cuffdiff). Cuffnorm (http://cole-trapnell-lab.github.io/cufflinks/cuffnorm) was then used to measure the fragments per kilobase per million mapped reads (FPKM) level for each gene. All the DEGs were collected for PCC calculation. Only the gene pair with a PCC value > 0.9 and a *P* value < 0.05 was considered the co-expressed partner.

### Transcription factor prediction

The protein sequence from the *A. paniculata* reference genome was used for TFs prediction via the iTAK program [26] workflow with the default parameters.

### Real-time quantitative PCR (qPCR)

Total RNA was extracted as described above. cDNA was synthesized by reverse transcription using the HiScript III RT SuperMix for qPCR (+gDNA wiper) kit (Vazyme) according to the manufacturer’s instructions. The qPCR assay was performed using the ChamQ Universal SYBR qPCR Master Mix kit (Vazyme) according to the manufacturer’s instructions. *ApACT* served as the internal gene for normalization, and the primer sequences used in the qPCR reaction are listed in Table S1.

### Virus-induced gene silencing (VIGS) experiment

The *ApTrihelix1* fragments were cloned into the *pLY156* (*pTRV2*) vector as the *pLY156:ApTrihelix1*. The constructed plasmids, including *pTRV1*, *pLY156:ApTrihelix1,* and the *pTRV2* empty vector, were transformed into *Agrobacterium tumefaciens* GV3101 (*pSoup*) and cultured at 28°C until the OD600 reached ~1. Then, the cells were resuspended in the infiltration buffer (10 mM MgCl_2_, 10 mM MES, pH 5.6, 100 mM acetosyringone) and adjusted to an OD600 of 0.2. *A. paniculata* at the four-leaf stage were infiltrated with the pTRV1/pTRV2 mixture using the vacuum infiltration method and grown for additional 10 days. Then, samples were used for AD measurement via HPLC or gene expression (qPCR) assay as mentioned above.

### Subcellular localization of ApTrihelix1

The CDS of *ApTrihelix1* were amplified using the high-fidelity enzyme KOD (TOYOBO) and cloned into the *pGreen-35S-GFP* (*C18*) vector to generate *35S: ApTrihelix1-GFP* vectors. The resulting vectors transformed into *Agrobacterium tumefaciens* GV3101 (*pSoup*) and were infiltrated into tobacco leaves. GFP fluorescence signals were then observed by a confocal microscope. The primers used for vector construction are listed in Table S1.

### ChIP-seq, data processing, and ChIP-qPCR assay

The *35S: ApTrihelix1-GFP* vector (mentioned above) was transiently expressed in the seedlings by the vacuum infiltration method described above. Samples were fixed with 1% formaldehyde, and ChIP was performed using the anti-GFP antibody (Abcam) and the ChIP-seq or the input library constructed by the Tn5 transposase according to previous reports [27] and sent for sequencing using the NovaSeq 6000 platform in the paired-end mode (PE150). For ChIP-seq data processing, adaptors and low-quality reads were removed by the TrimGalore (−nextera) software and then mapped to the *A. paniculata* reference genome [11] using Bowtie2 [28]. Mapping reads with mapping qualities (MAPQ) value over 30 were extracted by Samtools (-q 30) [29] and used for peak calling by MACS2 using default parameters [18]. Deeptools [30] and IGV software [31] were used for the visualization of the ChIP-seq data. HOMER (http://homer.ucsd.edu/homer) was used for peak annotation, and MEME [32] was used for motif scanning.

For ChIP-qPCR, ChIPed DNA from transiently transformed or mock plants were used. Primers (Table S1) were designed near the TF motif, and qPCR was performed using the ChamQ Universal SYBR qPCR Master Mix kit (Vazyme) according to the manufacturer’s instructions. Relative enrichment was calculated by comparing the signal in ChIPed samples to that in input samples. Values were then normalized relative to the signal obtained from the *ApACT*.

### Yeast One-Hybrid assay

The bait vector *pAbAi-pApCPS2* was from a previous study [9, 10]. The CDS of *ApTrihelix1* was cloned into the yeast expression vector *pGADT7*, yielding the effector vectors *pGADT7:ApTrihelix1* using the primer (Table S1). The *pAbAi-pApCPS2* was linearized with the restriction enzyme *Bst*BI and then transformed into competent Y1H gold yeast. Positive yeast with *pAbAi-pApCPS2* were inoculated onto SD/-Ura solid medium supplemented with varying concentrations of AbA for the selection of the best AbA concentration. Then, the constructed *pGADT7-ApTrihelix1* was transformed into yeast harbored with a *pAbAi-pApCPS2* fragment. Positive yeast transformants were selected by plating on SD/−Leu solid medium and then testing the interaction by adding AbA.

### Transient assay

For effector construction, *ApTrihelix1* or *ApTrihelix1:SRDX* was amplified from the cDNA by different primers (Table S1) and cloned into the *pGreenII 62-SK*. The reporter *pApCPS2:LUC* was from a previous study [9, 10]. The effectors and reporter were separately transformed into the competent *Agrobacterium tumefaciens* strain GV3101 (*pSoup*). Different combinations of the effector and reporter were mixed and then infiltrated into tobacco leaves using a 1 ml needleless syringe. The transformed leaf was sprayed with a reaction buffer containing 1 mM luciferin substrate, and the luminescence signal was observed via a plant in *vivo* imaging system (Tanon 5200, China). The TransDetect Double-Luciferase Reporter Assay Kit (TransGene) was used for luciferase activity quantification according to the manufacturer’s instructions. The Renilla (REN) luciferase activity was used for internal control.

## Supplementary Material

Web_Material_uhag118

## Data Availability

The RNA sequencing raw data have been submitted to the National Center for Biotechnology Information (NCBI) under the BioProject accession number PRJNA657334. The raw data of ApTrihelix1 ChIP-seq were submitted to the National Genomics Data Center (https://ngdc.cncb.ac.cn/, BioProject: PRJCA058555).
