# Supplementary material for: Light-responsive transcription factor ApTrihelix1 modulates andrographolide biosynthesis via targeting ApCPS2 in Andrographis paniculata
Source: Hortic Res. 2026 Apr 7;13(7):uhag118. doi: 10.1093/hr/uhag118 (PMC13341122; doi:10.1093/hr/uhag118)
Supplement: Web_Material_uhag118 [file web_material_uhag118.zip › SupFigs V1.docx]

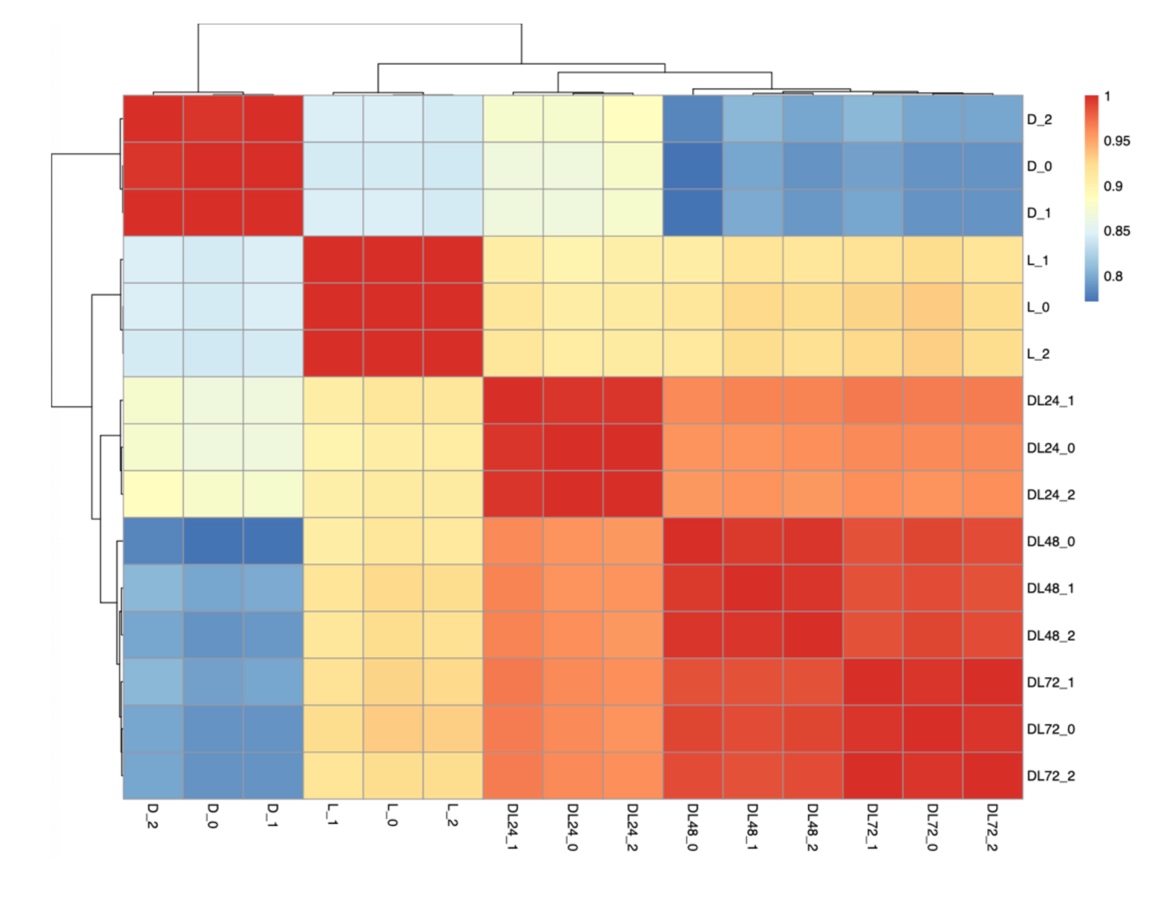


**Figure S1. The correlation among the light treated RNA-seq of *A. paniculata* seedlings.**


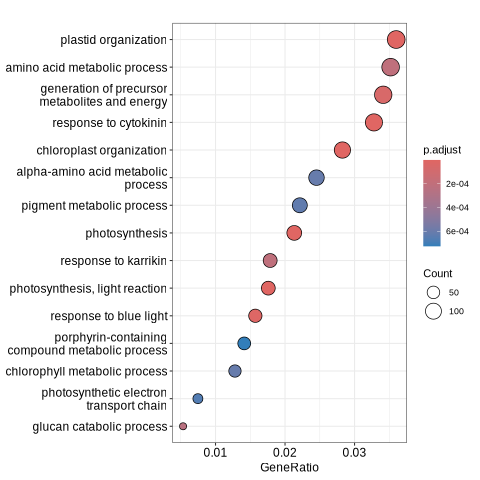


**Figure S2. GO enrichment analysis of all the differentially expressed genes in light treatment of *A. paniculata* seedlings.**

**
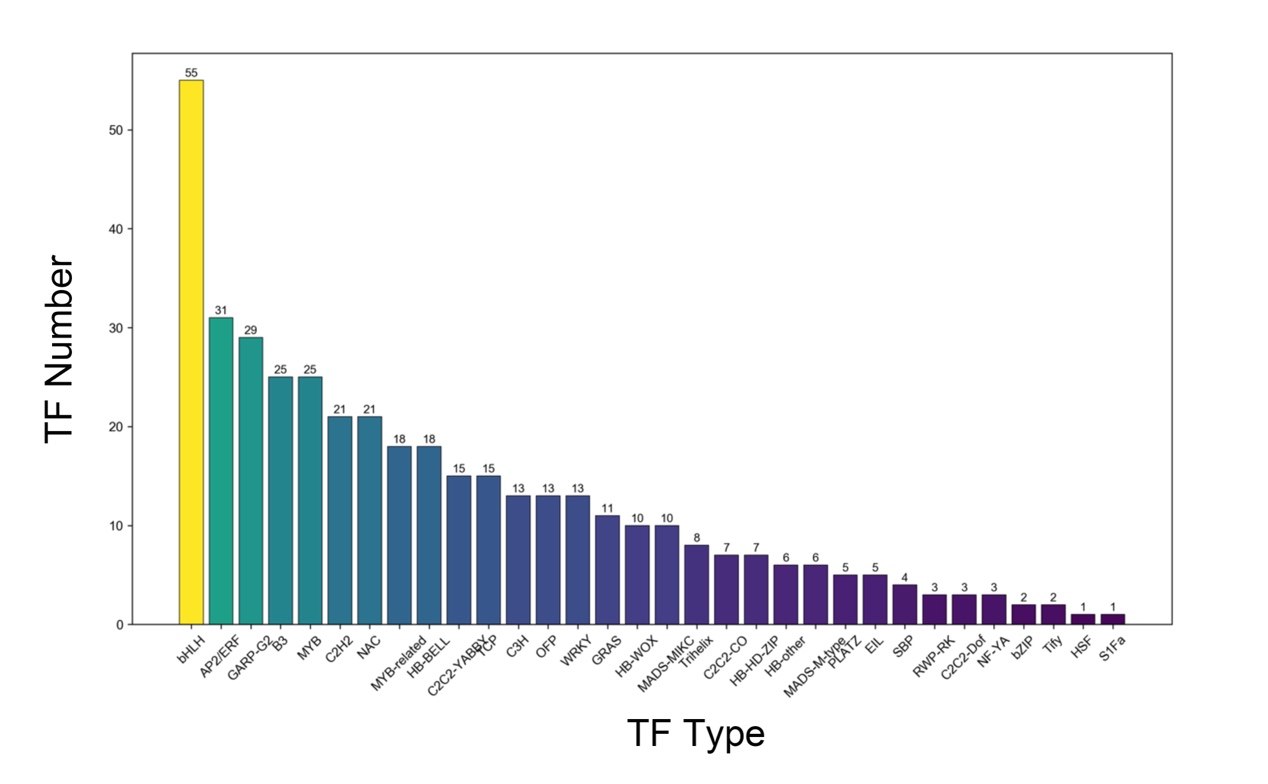
**

**Figure S3. The number of TF type co-expressed with the ADRGs.**


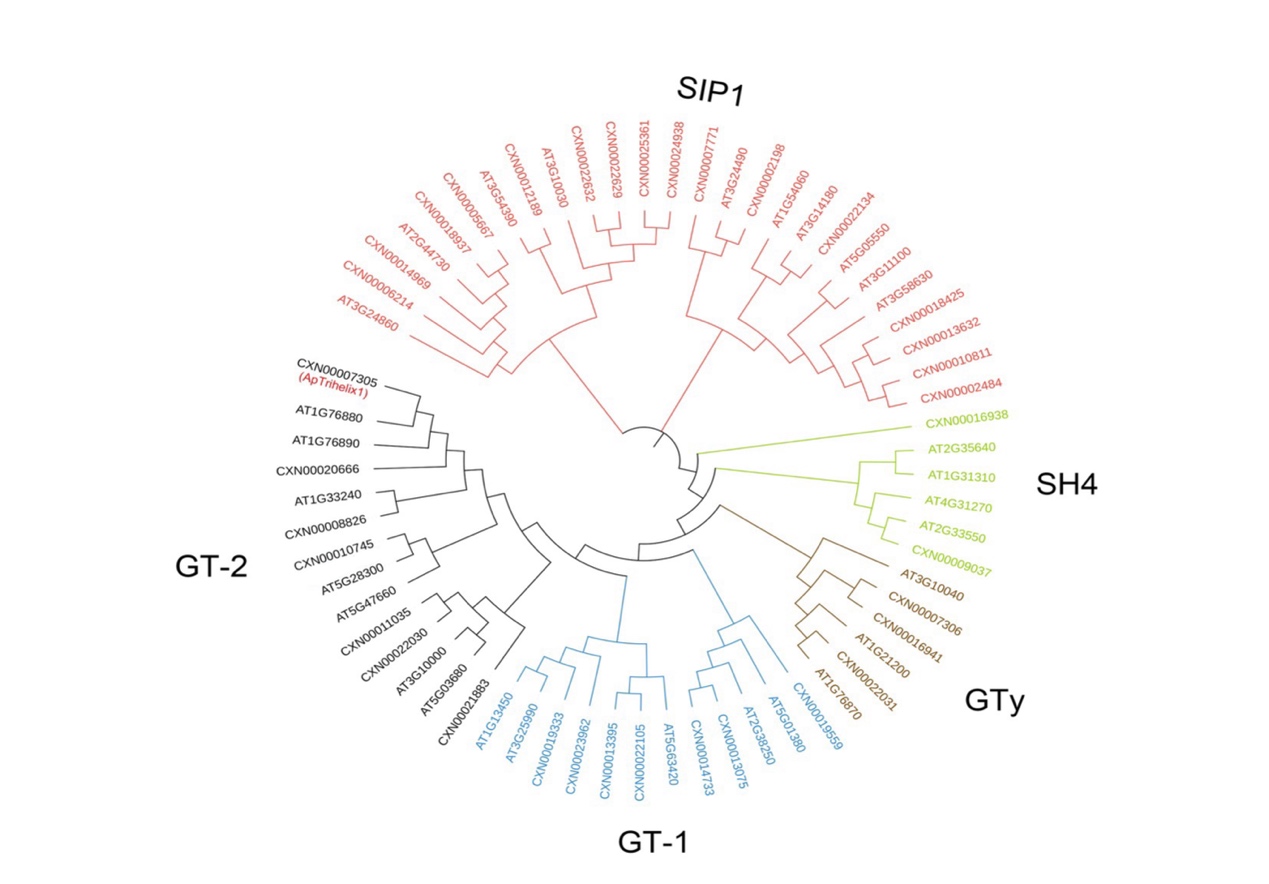


**Figure S4. Phylogenetic tree analysis of Trihelix transcription factors in genome of *A. paniculata*. The phylogenetic tree was constructed using the neighbor-joining (NJ) method.**


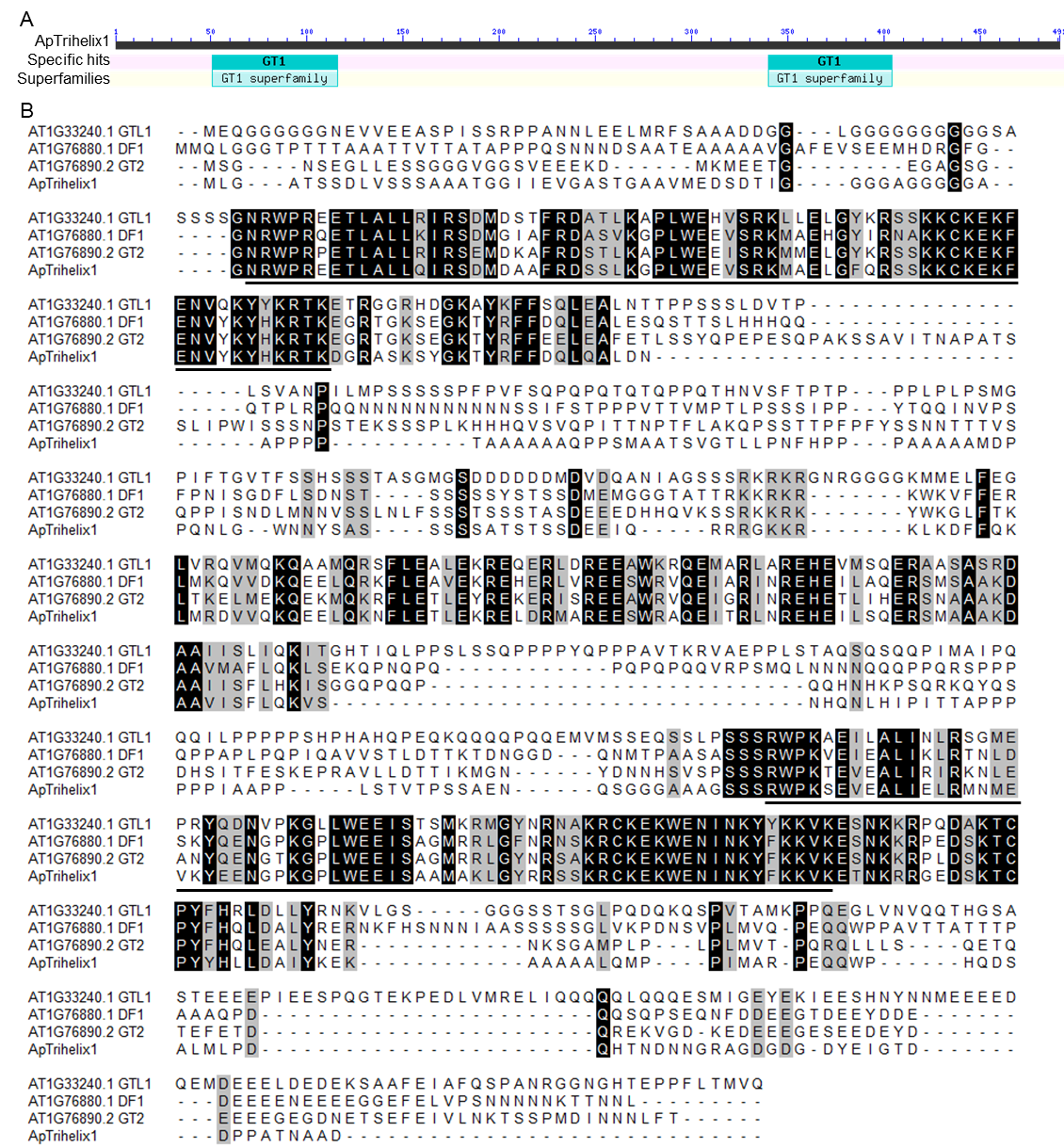


**Figure S5. Protein alignment of ApTrihelix1 and its homologous genes in *Arabidopsis thaliana*.**


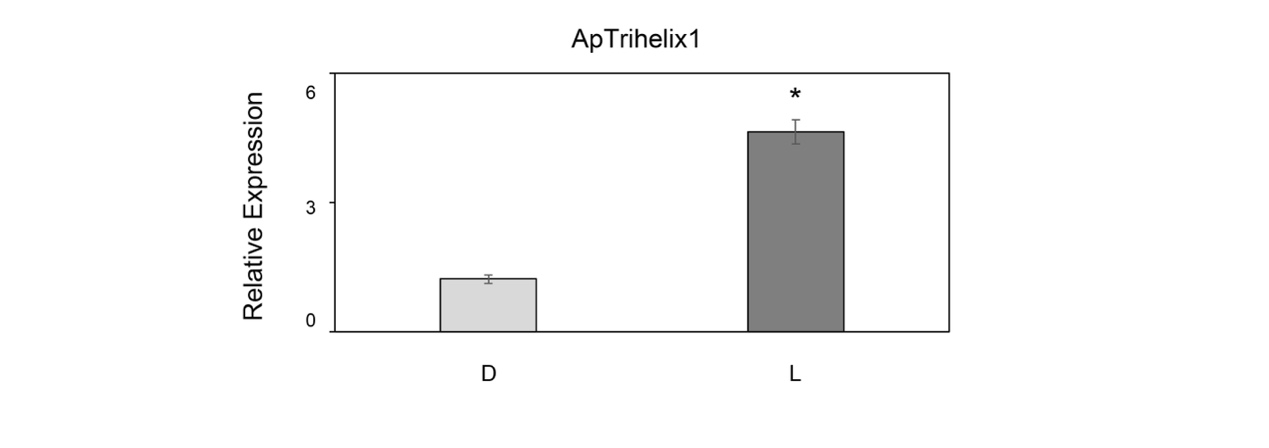


**Figure S6. Quantitative real-time PCR (qPCR) analysis of *ApTrihelix1* in light- (L) or Dark- (D) grown seedlings.** Asterisks (*) indicate significant differences between D and L (Student’s *t-*test, *p* < 0.05).


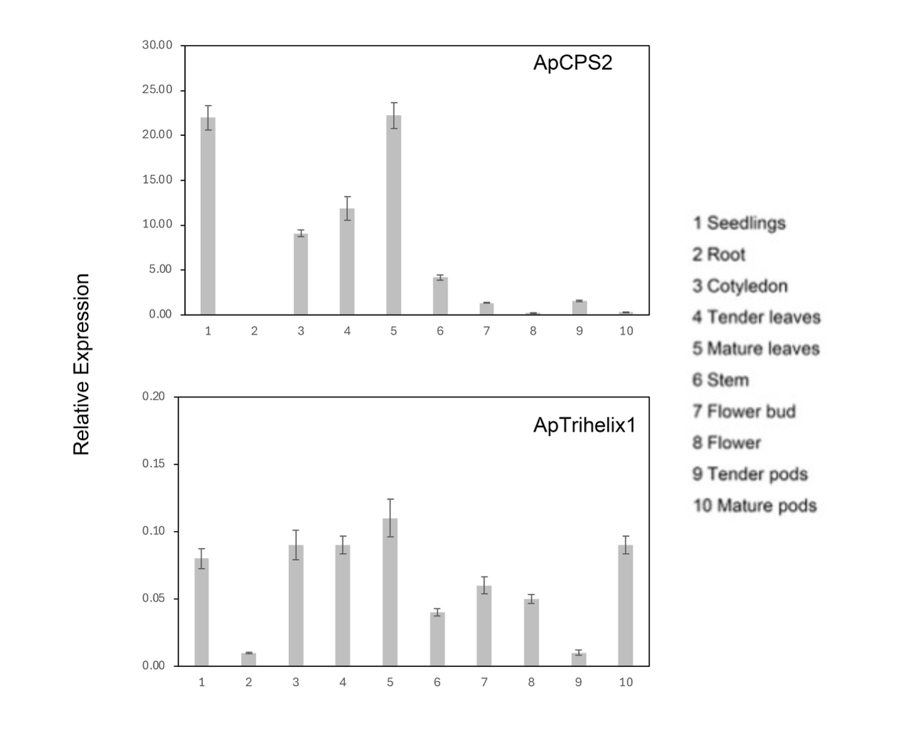


**Figure S7. Quantitative real-time PCR (qPCR) analysis of *ApTrihelix1* and *ApCPS2* in different tissues of *A. paniculata*.**

**
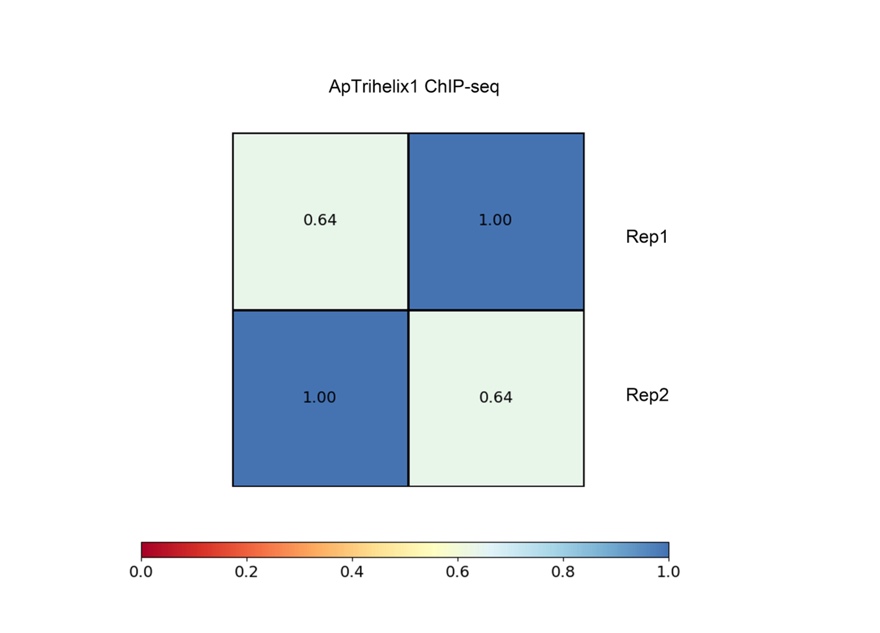
**

**Figure S8. The correlation between the two replicates (Rep1 and Rep2) of ApTrihelix1 ChIP-seq.**


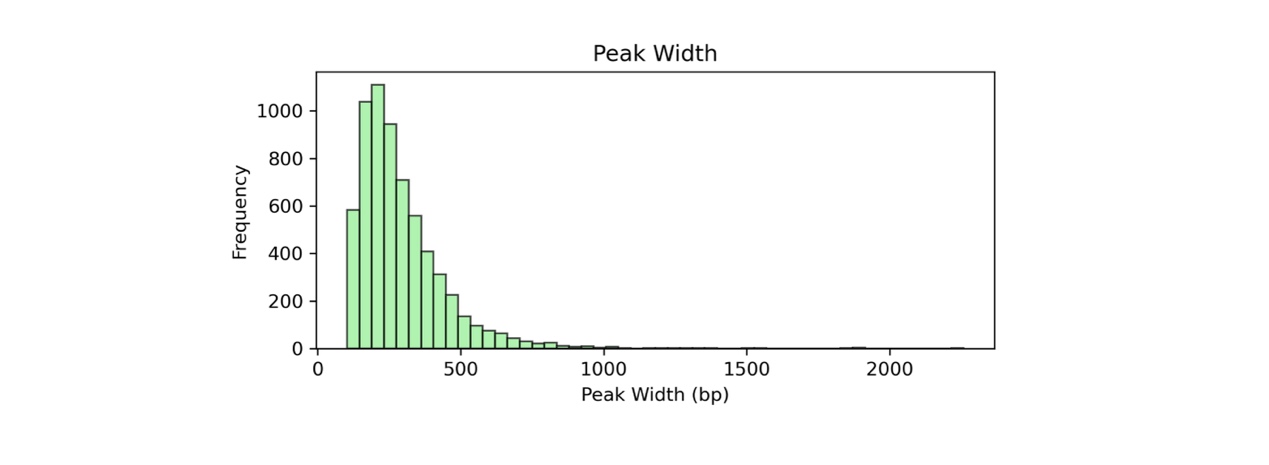


**Figure S9. The peak width of ApTrihelix1 enriched peaks.**


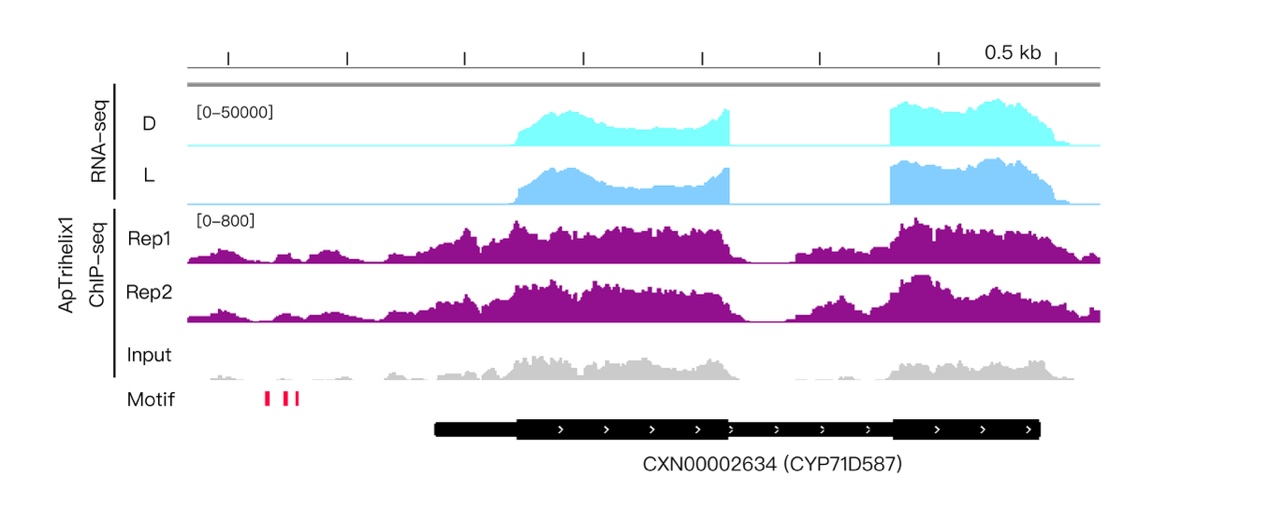


**Figure S10. IGV screenshot shows the enrichment of ApTrihelix1 ChIP-seq at the another ADRGs, CYP71D587 gene.** Expression of *CYP71D587* do not showed differences between light or dark-grown seedlings.


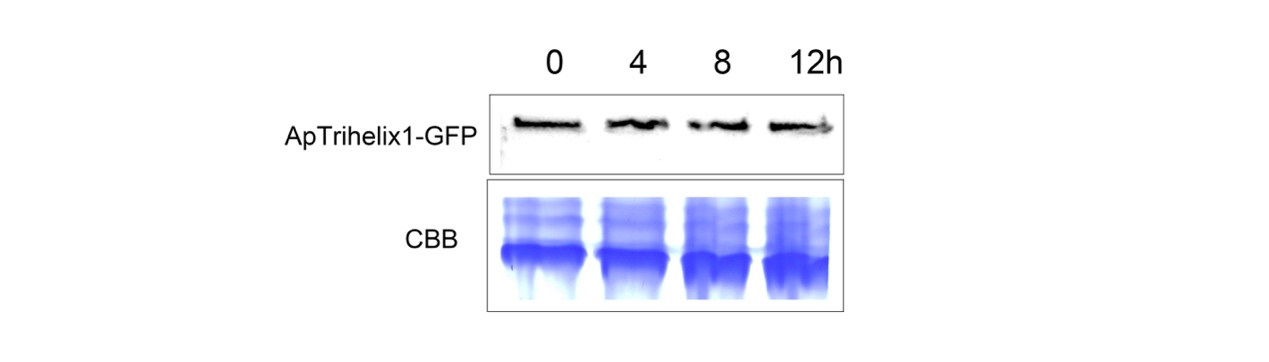


**Figure S11.Protein level of ApTrihelix1-GFP under dark treatment.** Plants transiently overexpressing ApTrihelix1-GFP were subjected to dark treatment for 0, 4, 8, and 12 hours, respectively. The protein level of ApTrihelix1-GFP was detected by western blot analysis using an anti-GFP monoclonal antibody. CBB (Coomassie Brilliant Blue) staining was used as a loading control to confirm equal protein loading across all lanes.
