# Supplementary figures and images for: Light-responsive transcription factor ApTrihelix1 modulates andrographolide biosynthesis via targeting ApCPS2 in Andrographis paniculata
Source: Hortic Res. 2026 Apr 7;13(7):uhag118. doi: 10.1093/hr/uhag118 (PMC13341122; doi:10.1093/hr/uhag118)

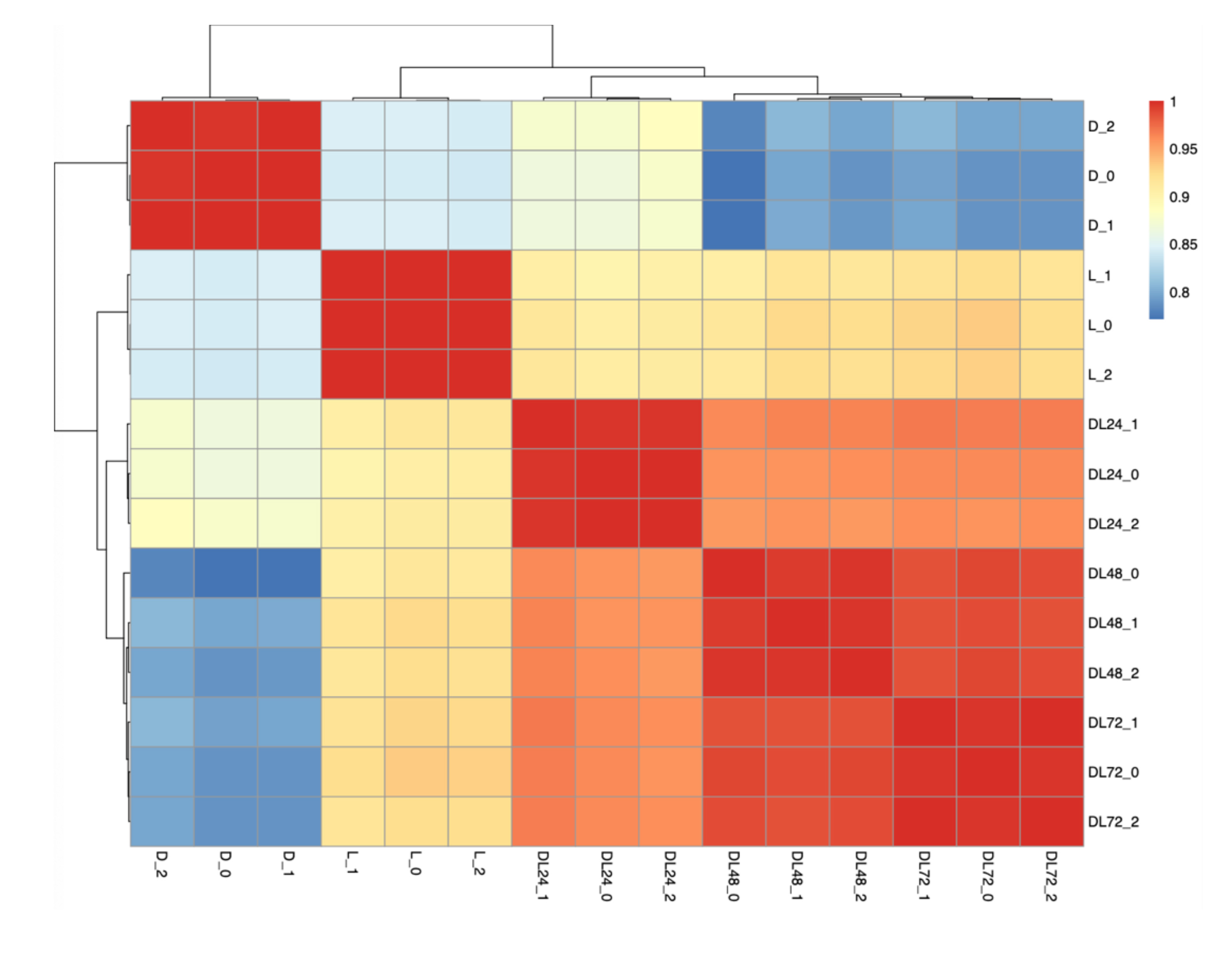

Supplement: Web_Material_uhag118 [file web_material_uhag118.zip › Figure S1.jpg]

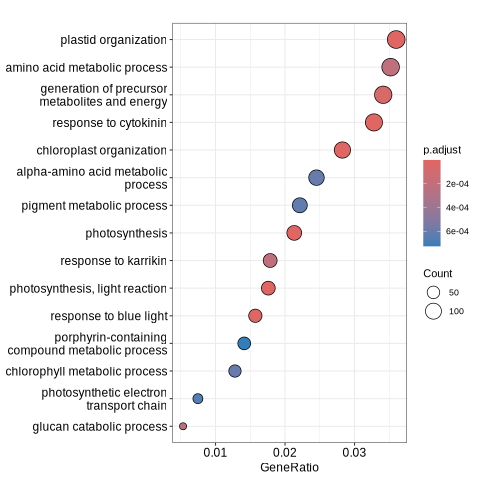

Supplement: Web_Material_uhag118 [file web_material_uhag118.zip › Figure S2.png]

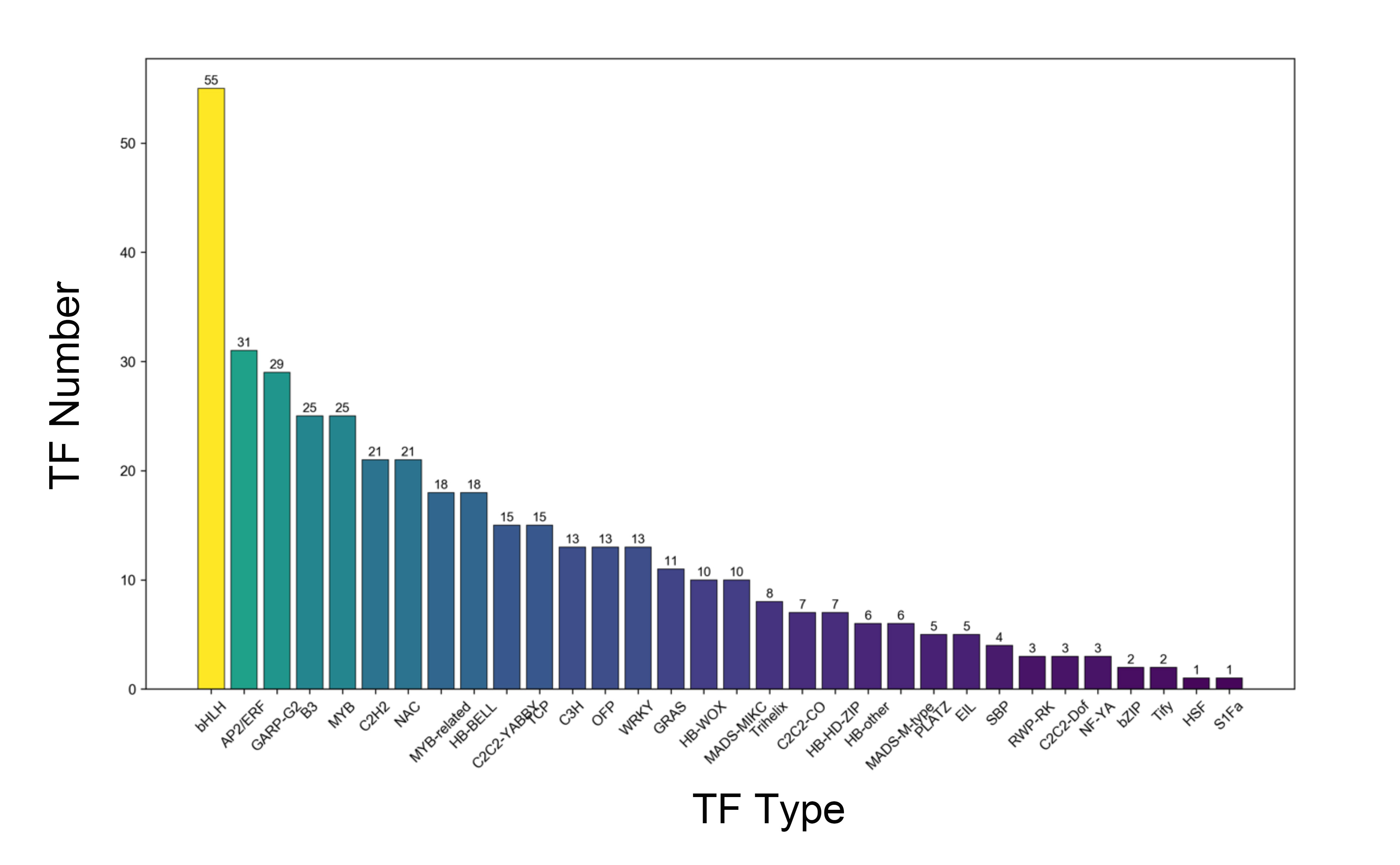

Supplement: Web_Material_uhag118 [file web_material_uhag118.zip › Figure S3.jpg]

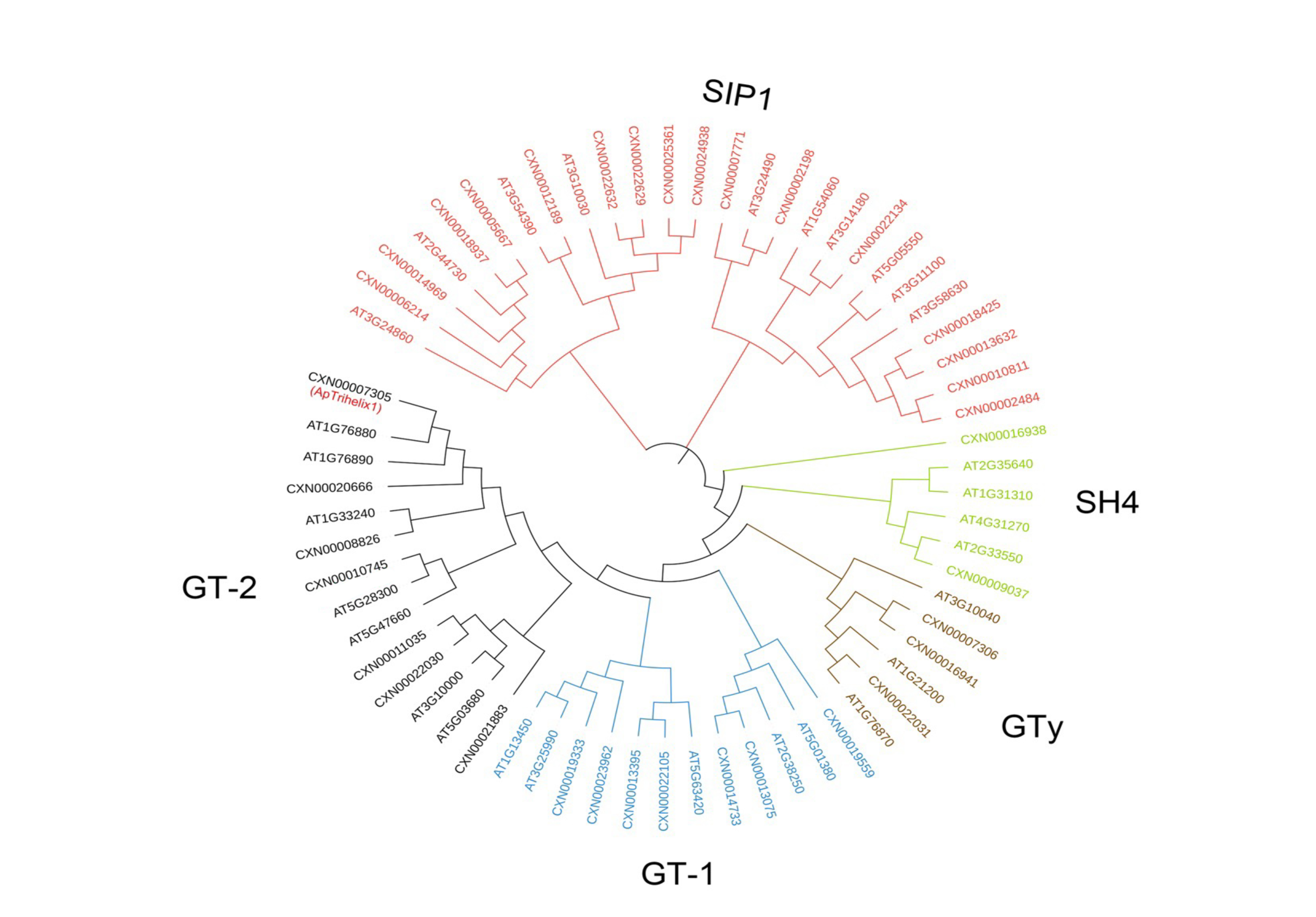

Supplement: Web_Material_uhag118 [file web_material_uhag118.zip › Figure S4.jpg]

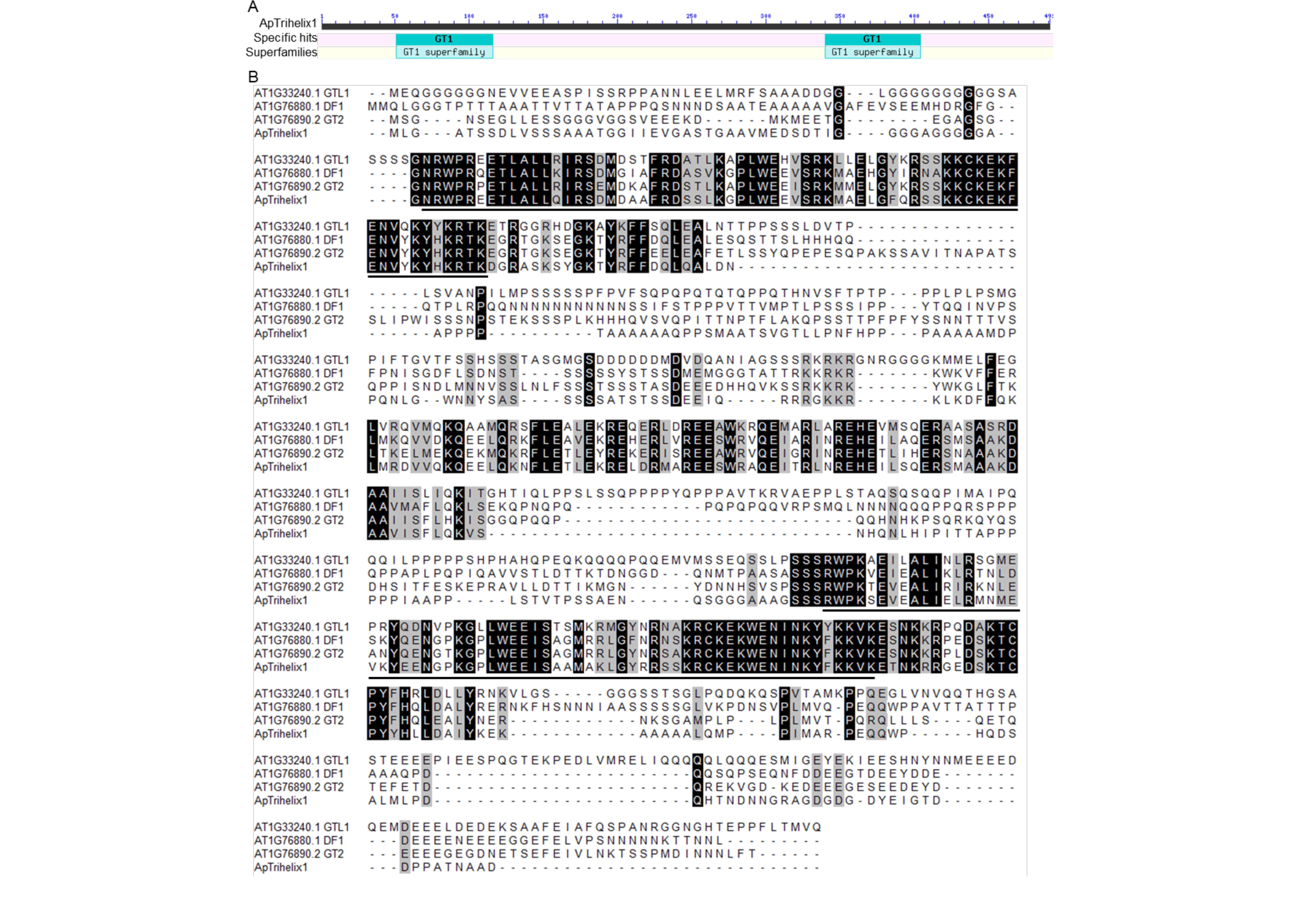

Supplement: Web_Material_uhag118 [file web_material_uhag118.zip › Figure S5.jpg]

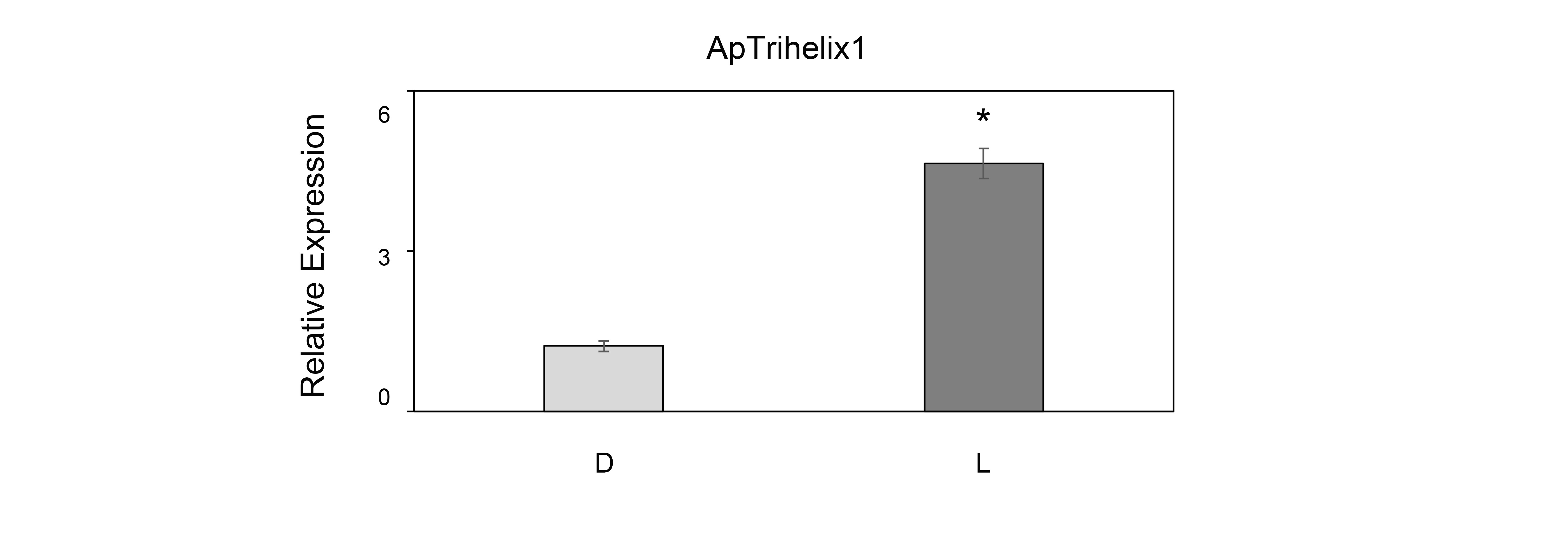

Supplement: Web_Material_uhag118 [file web_material_uhag118.zip › Figure S6.jpg]

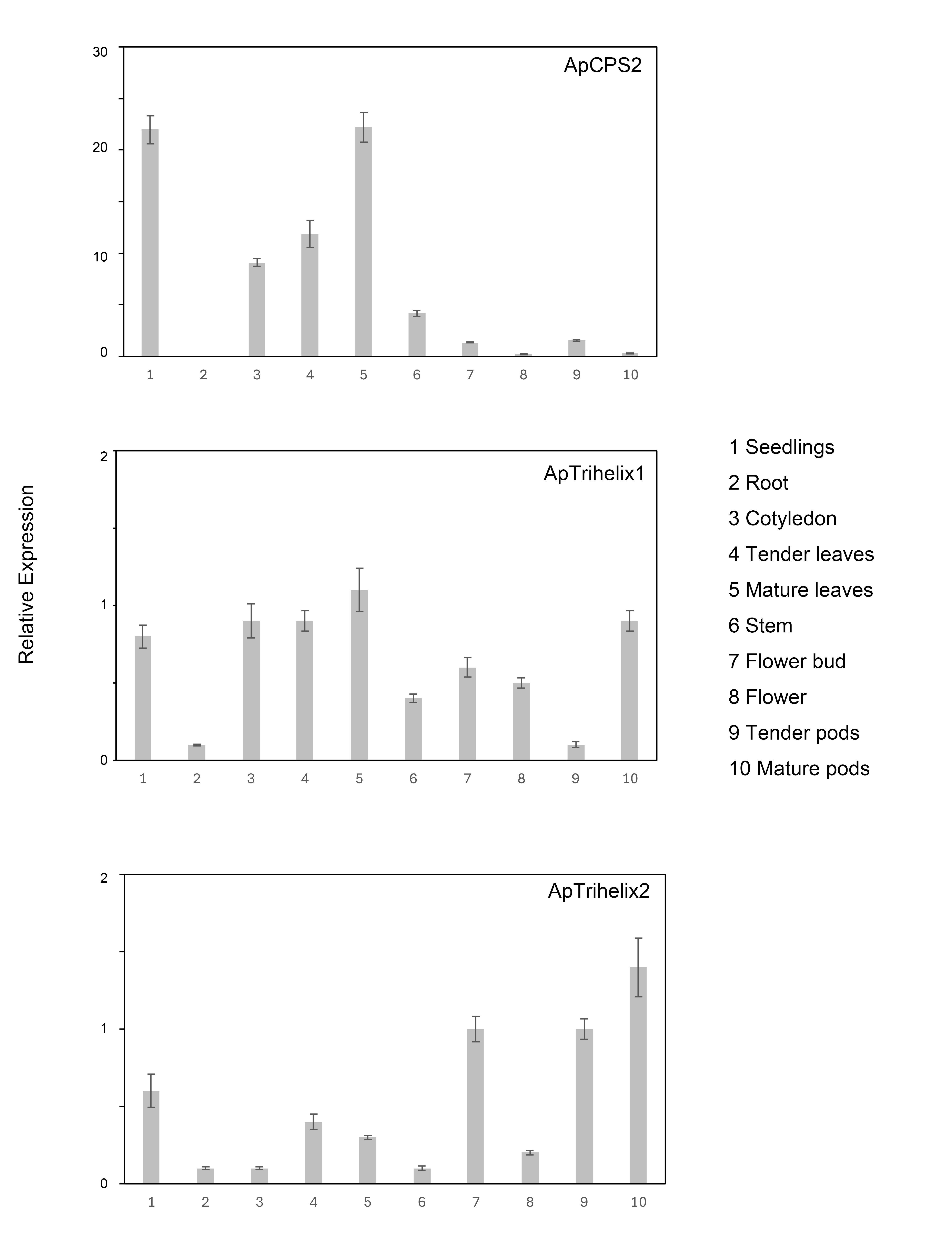

Supplement: Web_Material_uhag118 [file web_material_uhag118.zip › Figure S7.jpg]

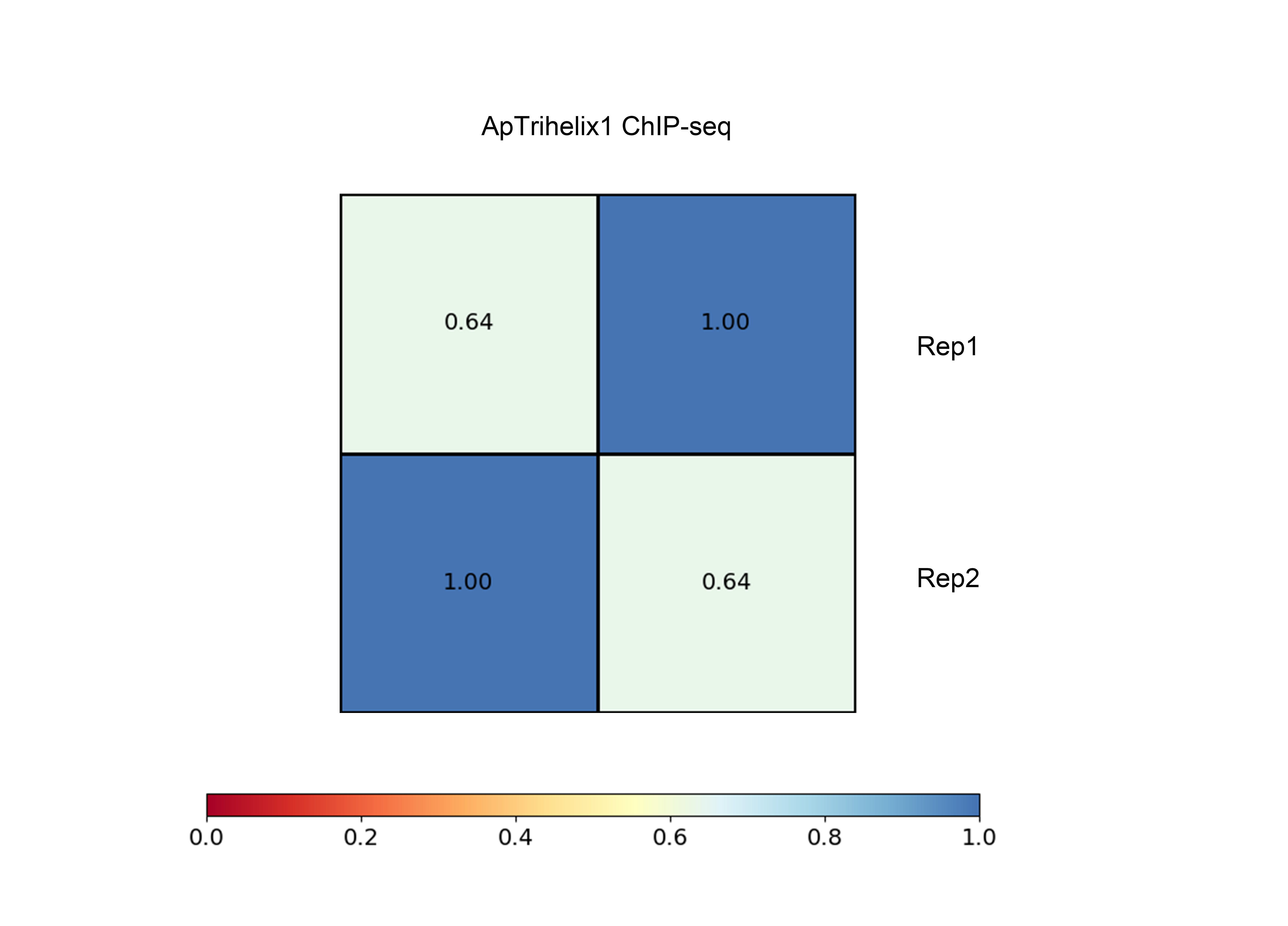

Supplement: Web_Material_uhag118 [file web_material_uhag118.zip › Figure S8.jpg]

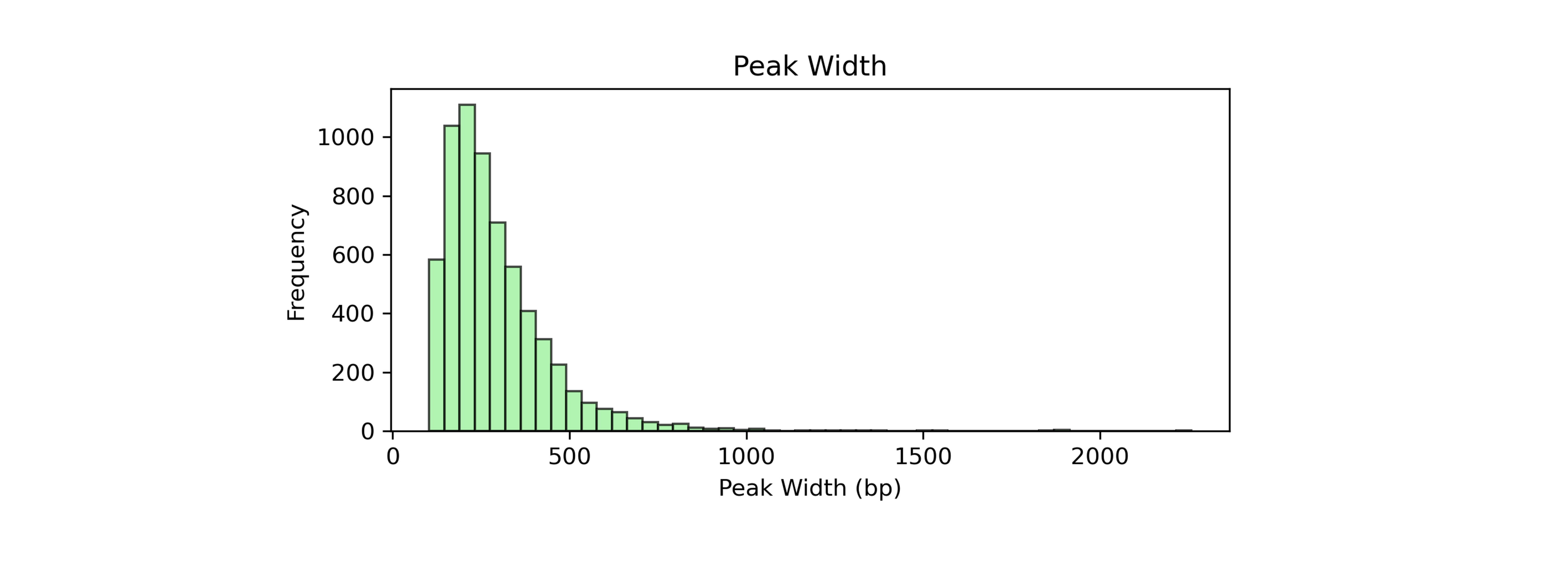

Supplement: Web_Material_uhag118 [file web_material_uhag118.zip › Figure S9.jpg]

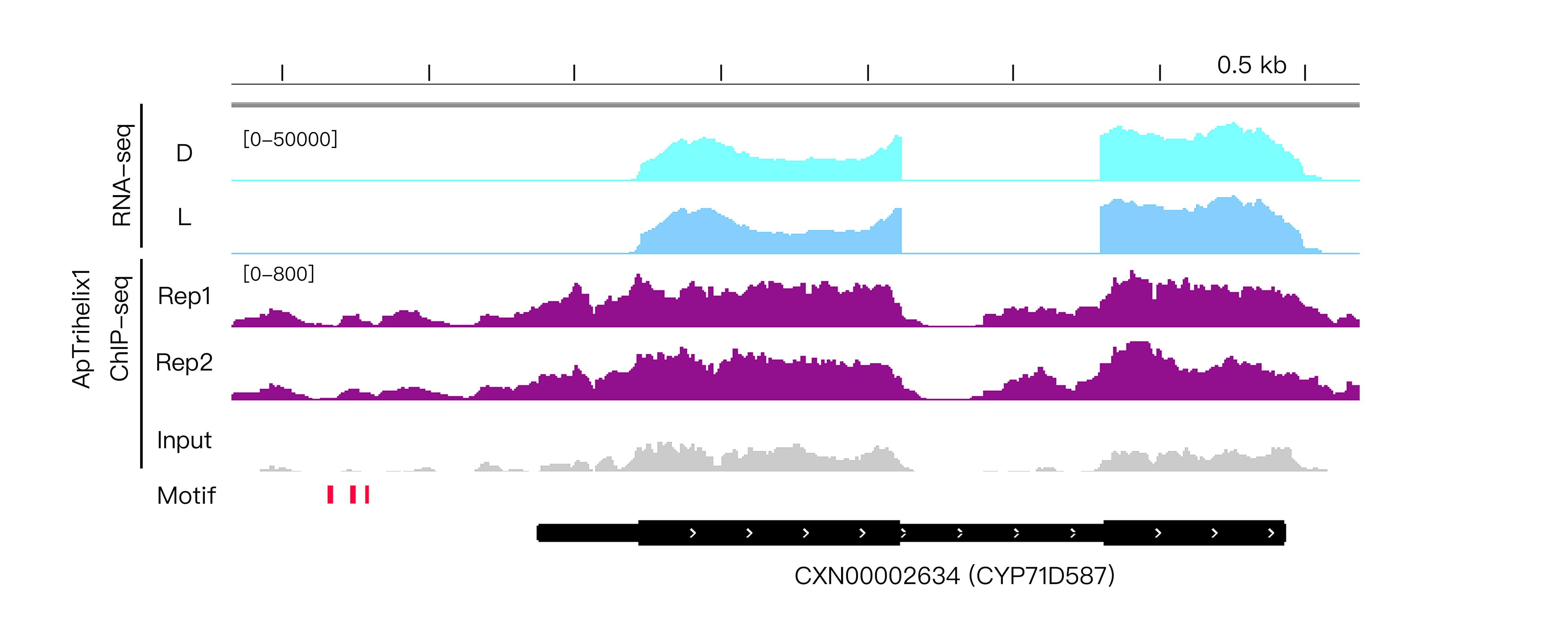

Supplement: Web_Material_uhag118 [file web_material_uhag118.zip › Figure S10.jpg]

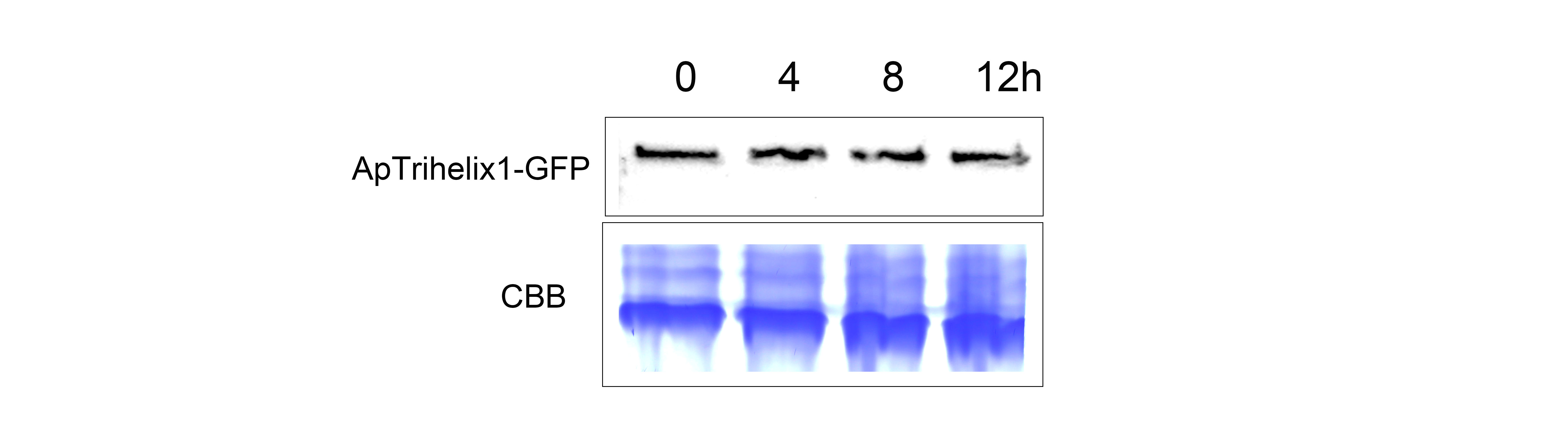

Supplement: Web_Material_uhag118 [file web_material_uhag118.zip › Figure S11.jpg]
